# Supplementary figures and images for: Chemical genomic guided engineering of gamma-valerolactone tolerant yeast
Source: Microb Cell Fact. 2018 Jan 12;17:5. doi: 10.1186/s12934-017-0848-9 (PMC5767017; doi:10.1186/s12934-017-0848-9)

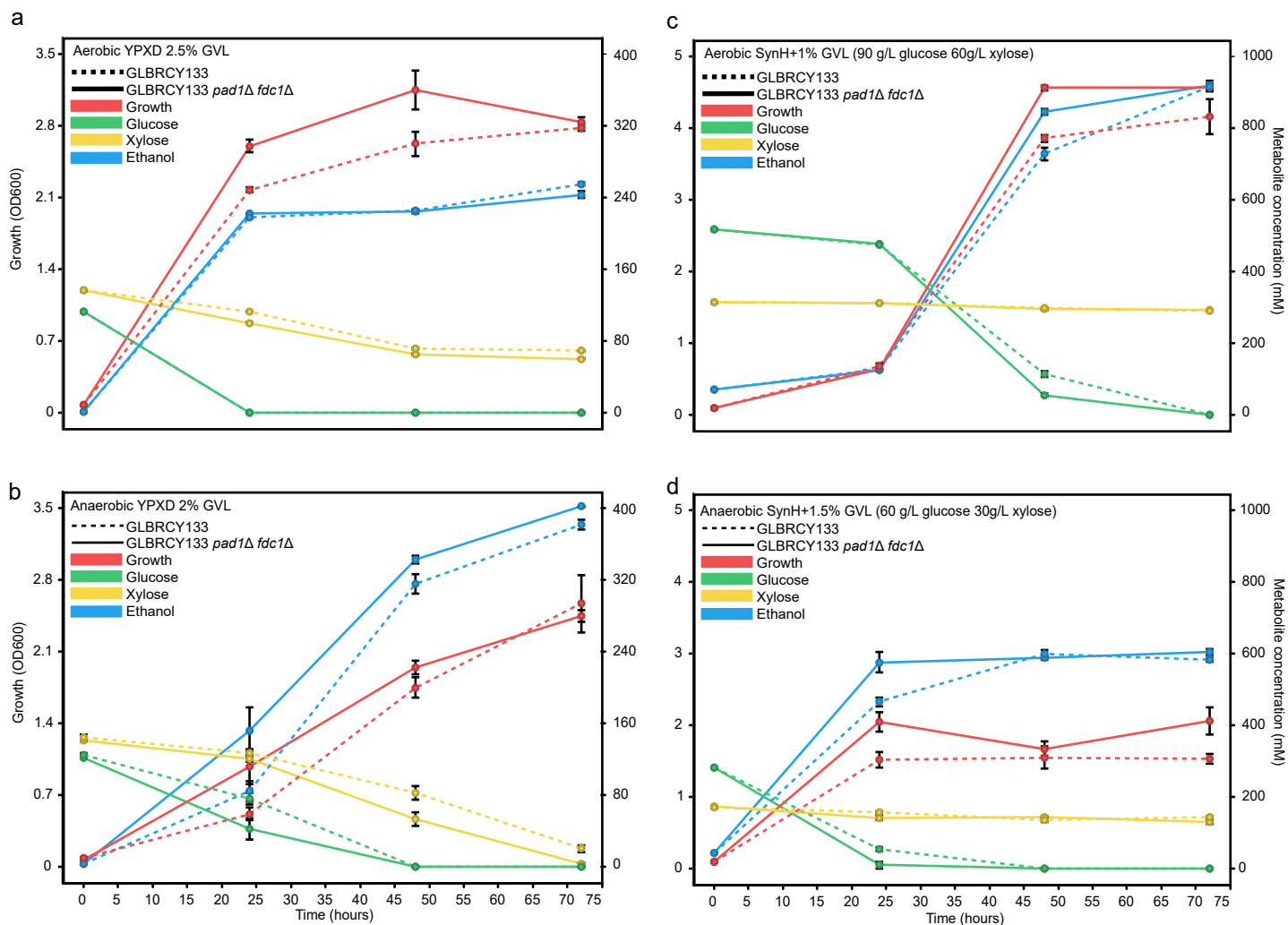

Supplement: Supplementary file 3 — Additional file 3. Growth, sugar consumption, and ethanol production of Y133 pad1∆ fdc1∆ vs Y133 grown in anaerobic and aerobic conditions with either rich media (YPXD) or synthetic hydrolysates (n = 3, Mean ± SE). [file 12934_2017_848_MOESM3_ESM.pdf]
